# Periodic Limb Movements Syndrome in Patients With Cerebral Small Vessel Disease: Protocol for a Prospective Observational Study

**DOI:** 10.3389/fneur.2021.700151

**Published:** 2021-09-27

**Authors:** Ekaterina Spektor, Ingo Fietze, Mikhail G. Poluektov

**Affiliations:** ^1^Department of Sleep Medicine, Chair of Neurology and Neurosurgery, University Clinical Hospital No. 3, I.M. Sechenov First Moscow State Medical University, Moscow, Russia; ^2^Center of Sleep Medicine, Charité - Universitätsmedizin Berlin, Berlin, Germany; ^3^The Fourth People's hospital of Guangyuan, Guangyuan City, China; ^4^The Federal State Autonomous Educational Institution of Higher Education, I.M. Sechenov First Moscow State Medical University of the Ministry of Health of the Russian Federation, Moscow, Russia

**Keywords:** aged, cerebral small vascular diseases, cognitive dyfunctions, magnetic resonace imaging (MRI), polysomnogaphy, periodic limb movement, sleep

## Abstract

**Background:** Cerebrovascular diseases are the leading cause of cognitive decline and dementia. Therefore, the investigation of the potential ways to slow down the disease progression is an important research field. Periodic limb movements in sleep (PLMS) are known to be associated with transient changes in heart rate and blood pressure. These changes might influence the course of cerebral small vessel disease (cSVD). Nevertheless, the clinical significance of PLMS, particularly its influence on cardiovascular diseases course, is still controversial and underinvestigated.

**Methods/design:** Patients from 60 to 75 years old diagnosed with cSVD will undergo nocturnal polysomnography. Subjects with apnea/hypopnea index under 5 will be enrolled. Sleep quality and daytime functioning will be assessed at baseline with self-reported questionnaires. Brain MRI and cognitive assessment will be performed at baseline and in the 2-year follow-up. Progression of cSVD markers and cognitive dysfunction will be compared between patients with PLMS index (PLMI) equal to or more than 15 movements per hour of sleep and controls (PLMI <15/h).

**Discussion:** The negative role of PLMS in cSVD progression and related cognitive decline is expected. We suppose that patients with PLMS tend to worsen in cognitive performance more rapidly than age-, gender-, and comorbidity-matched controls. We also expect them to have more rapid white matter hyperintensities and other cSVD marker progression. The limitations of the study protocol are the short follow-up period, the absence of a treatment group, and inability to make a conclusion about causality.

## Introduction

### Background

Cerebrovascular diseases (CVD) remain a global problem today because of high prevalence and related cognitive impairment. Increase in vascular dementia rate is the current trend worldwide. Thus, the identification of new predictors for CVD seems to be an important medical and social problem. Cerebral small vessel disease (cSVD) is one of the most prevalent forms of CVD. This term is used to describe clinical features and structural changes in brain tissue occurring due to damage of small perforating arterioles as well as capillaries and venules. This vascular pathology results in white tissue and basal ganglia lesion that leads to neurological deficit and cognitive decline (1). cSVD is considered to be the leading cause of cognitive dysfunction and dementia (2–4). Moreover, cSVD is associated with an increasing risk of cerebral infarct and intracranial hemorrhage (5, 6). However, cSVD is a broad term, and it encompasses “pure cSVD,” Binswanger's disease, and mixed dementia (when Alzheimer's disease overlaps with cSVD), which are inherited forms of cSVD (7).

Although there are generally accepted risk factors for cSVD including hypertension, diabetes, and aging (1), recent researches have demonstrated a significant role of sleep disturbances in cSVD progression. The significance of obstructive sleep apnea is now well-established and extensively reported (8, 9). Nevertheless, there is a growing body of literature that recognizes the negative influence of other sleep disorders, including periodic limb movements (LMs) in sleep (PLMS), on the course of cardiovascular diseases and cSVD in particular. A probable explanation of this impact is a sleep fragmentation that results in episodes of heart rate increase and blood pressure elevation (8–12).

PLMS is a condition characterized by periodic episodes of repetitive and stereotypic flexions of the hips, knees, and ankles (less common—arms movements) that manifests during the sleep. Diagnosis of periodic limb movements disorder (PLMD) requires conformity with two criteria. Firstly, periodic limb movements index (PLMI) >5/h in children and >15/h in adults must be detected by polysomnography (PSG). Second, PLMS in sleep must lead to clinically significant sleep disturbance or impairment in different relevant areas of functioning and cannot be better explained by another current sleep, medical, or neurological disorder such as narcolepsy, restless leg syndrome (RLS), or REM sleep behavior disorder (13, 14). The prevalence of PLMS worldwide, including the Russian population, is estimated to be 3.9–6% in the general population and around 34% in aged patients (above 60 years) (15, 16).

PLMS are described to be associated with various conditions, such as congestive heart failure (17), alcohol abuse (18), Tourette syndrome (19), syringomyelia (20), multisystem atrophy, and Parkinson's disease (21). Increased PLMS are also observed in a significant proportion of patients with various sleep disturbances (narcolepsy and obstructive sleep apnea). Moreover, this phenomenon is also detected in healthy subjects, and the prevalence increases with age (13, 16, 22). The closest association of PLMS is found with RLS. Approximately 85% of patients with RLS show high PLMI during nocturnal sleep (13, 22).

### Pathophysiology and Cardiovascular Effects of PLMS

Initially, PLMS were studied in close connection with RLS. These conditions are supposed to share common pathophysiological mechanisms since E. Lugaresi et al. showed that most RLS patients had periodic leg movements during sleep (23, 24). RLS is defined by the International Classification of Sleep Disorders (III Edition) as the syndrome characterized by an urge to move the legs, sometimes accompanied by an uncomfortable sensation that occurs primarily with rest/inactivity; it is partially or totally relieved by movement, for as long as the movement occurs, and occurs primarily in the evening or at night (14). Despite close relations between PLMS and RLS, they are considered to be different neurological processes since one of them can occur in the absence of the other (23), although the precise origin of PLMD and RLS is not established. Probably, dopamine or its derivatives play the primary role in these states because a high prevalence of both RLS and PLMS is observed in Parkinson's disease patients (25). Moreover, worsening of symptoms is observed after dopamine antagonist intake and vice versa; remarkable improvement can be achieved by dopaminergic agent intake (26). On the other hand, Manconi et al. investigated the role of dopamine using pharmacological agents and revealed that RLS can be reduced using both dopaminergic and non-dopaminergic agents, whereas PLMS respond to dopaminergic agents only (27). Thereby it is likely, that PLMS is under stronger dopaminergic control in comparison with RLS.

The dysfunction of descending inhibitory pathways with subsequent disinhibition of the segmental apparatus of the spinal cord is believed to be the reason for PLMS (28). The D2 and D3 receptors on the preganglionic neurons of the lateral horns are considered to be involved (29). Probably, the diencephalic dopaminergic structures (A11 neuronal group) take part in the genesis of PLMS and RLS because the A11 diencephalospinal pathway is crucial for pain control and sensorimotor integration at the spinal level (30). For example, the study on rats showed the presence of A11 dopaminergic terminals in posterior spinal horns and their vicinity to the motor neurons of anterior horns (31). The model involving the crucial role of dopamine could explain the positive effect of intake of dopaminergic agents in patients with both PLMS and RLS.

The subcortical origin of PLMS is confirmed by the experiment with paired-pulse transcranial magnetic stimulation (ppTMS). Rijsman et al. revealed decreased intracortical inhibition and increased intracortical facilitation while the cortical excitability was normal in subjects with RLS and PLMS. These aberrations appeared more significant in the leg that was more disturbed with sensorimotor symptoms and had higher PLMI than the other leg (28). The presumption about the supraspinal subcortical origin of PLMS is confirmed by studies in patients with cervical or thoracic traumatic complete spinal cord injury. They discovered higher PLMI in comparison with healthy controls. The probable explanation is the damage of descending pathways and loss of the supraspinal inhibitory influence (32, 33). It is also confirmed indirectly by the investigation of H-reflex recovery curve in PLMD patients. The results of this study demonstrated increasing late facilitation and decreasing late inhibition in comparison with controls (28). These findings might reflect decreased postsynaptic inhibition.

There is evidence that PLMS provokes a considerable increase in sympathetic activity throughout sleep (34, 35), which results in heart rate and blood pressure increase (9, 36). The repeated increase in nocturnal heart rate and blood pressure are considered to be the reason for the negative influence of PLMS on the cardiovascular system. Nevertheless, some researchers deny the existence of persistent sympathetic hyperactivity in the absence of movement (in other words, sympathetic hyperactivity exists only when PLMS occurs) (37, 38). On the other hand, other authors highlighted the primary role of the sympathetic nervous system in the generation of PLMS (39). Thus, there is no consensus on whether the PLMS itself can change the autonomic tone.

PLMS is often associated with EEG arousals. It is not yet established whether one phenomenon is the reason for the other, or both phenomena have the same origin. However, the latter seemed more likely (22, 40). The effect of arousals on daytime consequences is controversial. The high frequency of PLMS with arousals during sleep could be a hypothetical trigger for nocturnal arrhythmias and hypertension (41), and PLMS-related arousals may be a risk factor of ventricular arrhythmia (42). However, the majority of the researchers do not emphasize the difference between the PLMS with arousals and the PLMS without arousals. There are data showing that PLMS frequency (with and without arousals) is associated with a significant increase in systolic blood pressure (10, 43).

However, evidence of the relationship between PLMS and cSVD is scarce. The prevalence of PLMS in the cSVD subpopulation was not evaluated. Coelho et al. showed that patients with a history of stroke had a greater prevalence of PLMS and significantly higher mean PLMI than controls (44), but according to the research made by Manconi et al., PLMS are equally frequent in patients with a history of stroke or TIA and the general population (45). There are data about a higher prevalence of RLS in patients with cardiovascular diseases (7.7–36%), which is three times higher than the prevalence of RLS in the general population (46). The cardiovascular effects of RLS are suggested by some researchers to be mediated by increased PLMI (47).

Current evidence suggests that there is repetitive sympathetic activation accompanying PLMS during night sleep. This phenomenon could be a potential link between PLMS and negative impact on the cardiovascular system (36). The presumed cardiovascular effects of PLMS could underlie reported associations of higher PLMI with arterial hypertension severity (48), the incidence of cardiovascular events (49), increased risk of death in patients with systolic dysfunction following acute decompensated heart failure (50), and mortality risk in patients with systolic heart failure (12). It is reasonable to assume that PLMS-related activation of the sympathetic nervous system could be an explanation of the negative role of PLMS in CVD course, although it remains an underinvestigated issue. It is shown in cross-sectional studies that the severity of PLMS is associated with the extent of white matter hyperintensities (WMH) in patients with cSVD (51, 52) and in patients with first-ever minor stroke or transient ischemic attack (53). On the other hand, it is known that any pathological state accompanied by sleep fragmentation (sleep apnea, PLMS, and insomnia) may increase the risk of vascular diseases (9). Therefore, a more general process could be responsible for increased cardiovascular risk in PLMS patients.

Another unresolved question is the relevance of PLMS to cognitive dysfunction. Whereas, PLMS could worsen the course of cSVD, the greater cognitive decline of PLMS patients is expected. However, only a few studies focused on this issue, and their results suggest a positive correlation between the PLMI and cognitive decline. Significant association of higher PLMS frequency with impaired executive function was demonstrated among older men without dementia (54), patients with Parkinson's disease independent from psychomotor speed (55), and patients with cSVD (52). Besides, worse sleep efficiency has been also shown to be associated with poorer performance in tasks evaluating the executive domain (56), but the influence of PLMS on sleep quality and its ability to lead to sleep fragmentation is disputable. Moreover, some authors report that age-related changes in sleep architecture with a decline in slow-wave sleep might mediate age-related decline in executive control (57). It appeared confusing since the prevalence of PLMS increases with age too.

On the contrary, the cognitive impairment in cSVD is fully investigated, and it involves similar changes in executive functions, attention, language fluency, and set shifting (58, 59). MRI features of cSVD are summarized in STRIVE criteria (STandards for ReportIng Vascular changes on nEuroimaging). They consist of WMH, recent small subcortical infarcts, lacunes, cerebral microbleeds, and perivascular spaces (4). Substantial contribution to cognitive decline has been shown for frontal- and temporal-located cerebral microbleeds (60), progression of periventricular WMH (61), accumulation of lacunar infarctions (58), and perivascular spaces in combination with other markers (62).

### Rationale

The determination of the role of PLMS in the progression of cSVD MRI features and increase of cognitive dysfunction would create a new approach to patient management. Confirming of the negative impact of PLMS on the course of cSVD makes considerable further investigation the utility of the pharmacologic correction of PLMS in future studies. Currently, there is no generally accepted point of view on clinical significance of PLMS and its exact connection with both cSVD course and cognitive decline.

### Hypothesis

PLMI equal to or more than 15 movements per 1 h of sleep is a predictor of small vessel disease progression.

Null hypothesis: the presence of PLMS with index ≥ 15/h has no influence on cSVD course.

Alternative hypothesis (one sided): the presence of PLMS with index ≥ 15/h worsens cSVD course.

### The Aim and Objectives

The aim of the study was to define the role of PLMS in cSVD progression and related cognitive decline progression.

The main objectives of the study are:

1) Assessment of the cognitive function in small vessel disease patients at baseline and in the 2-year follow-up.
2) Assessment of MRI markers of cSVD at baseline and in the 2-year follow-up.
3) Assessment of sleep quality and health-related quality of life in patients with PLMS in comparison with those without PLMS.
4) Evaluation of PLMS impact on cSVD progression through constructing multiple regression model.
5) Assessment of the association between PLMS and poor sleep quality based on questionnaire data.

## Methods and Design

### Protocol Outline

This study was designed as a prospective observational study. It will be conducted in Moscow, Russia (Department of Neurology, I.M. Sechenov First Moscow State Medical University).

In-patients aged 60–75 diagnosed with cSVD according to current diagnostic criteria and neuroimaging standards (51, 54) will be evaluated for MRI markers of cSVD and cognitive function over a period of 2 years. General assessment of risk factors for cardiovascular disease will include identification of hypertension (office measured off medication conventional blood pressure of at least 140/90 mmHg), measuring total cholesterol level, calculation of body mass index, as well as revealing by self-report and exploring the medical history the presence of ischemic heart disease, diabetes, and heart rhythm disturbances. Additionally, the duration of smoking and habitual alcohol consumption will be assessed. For the latter purpose, a validated Russian version of the Alcohol Use Disorders Identification Test (AUDIT) will be applied (58).

**Figure 1:**
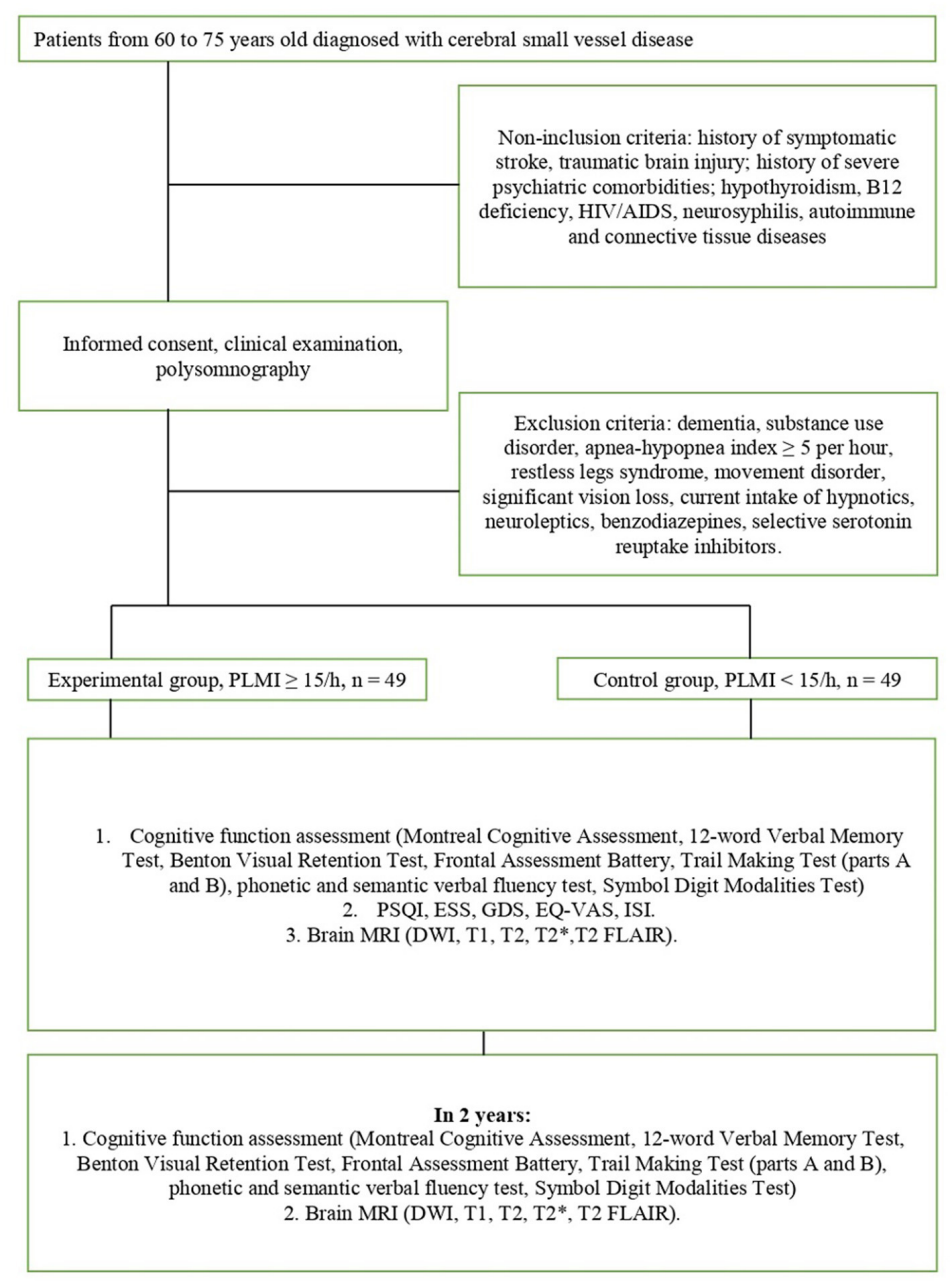
Design of the study.

The design of the study is schematically depicted in Figure 1. All enrolled patients will undergo overnight PSG at baseline. Based on PLMI, all patients will be divided into the experimental (PLMI ≥15/h) and control groups (PLMI <15/h). The assessment of the night sleep and quality of daytime functioning will be made at baseline only. Assessment of cognitive function and brain MRI will take place at weeks 0 (baseline) and 104 (2-year follow-up). The details on the applied study techniques and evaluation of night sleep, cognitive function, and MRI are given in parts 2.4, 2.5, and 2.6, respectively. The examination protocol is resumed in Table 1.

**Table 1:** Examination protocol.

**Methods**	**Baseline**	**2-years follow-up**	**Unit of measurement**	**Range**
Polysomnography	TST	None	Min	Not defined
	SOL	None	Min	Not defined
	WASO	None	Min	Not defined
	SE	None	%	0–100
	AHI	None	Number per hour	Not defined
	LMs	None	Number during the sleep	Not defined
	LMI	None	Number per hour	Not defined
	PLMI	None	Number per hour	Not defined
	PI	None	None	0–1
	SILMS index	None	Number per hour	Not defined
	PLMWI	None	Number per hour	Not defined
	PLMAI	None	Number per hour	Not defined
Questionnaires	AUDIT	None	Points	0–40
	PSQI	None	Points	0–21
	ESS	None	Points	4–24
	GDS	None	Points	0–30
	EQ-VAS	None	Points	0–100
	ISI	None	Points	0–28
Cognitive assessment	MoCA score	MoCA score, percentage change	Points, %	0–30
	12 word test total score	12 word test total score, percentage change	Points, %	0–24
	BVRT score	BVRT score, percentage change	Points, %	0–10
	FAB score	FAB score, percentage change	Points, %	0–18
	TMT-A time	TMT-A time, percentage change	sec, %	Not defined
	TMT-B time	TMT-B time, percentage change	sec, %	Not defined
	B/A ratio	B/A ratio, percentage change	None, %	Not defined
	SDMT score	SDMT score, percentage change	points, %	not defined
	PVFT	PVFT, percentage change	Words, %	Not defined
	SVFT	SVFT, percentage change	Words, %	Not defined
Brain MRI	WMH volume	WMH volume, percentage change	ml, %	Not defined
	Fazekas Scale	Fazekas Scale, percentage change	points, %	0–6
	Scheltens Scale	Scheltens Scale, percentage change	points, %	0–84
	cSVD Scale	cSVD Scale, percentage change	points, %	0–4
	None	Rotterdam progression scale	Points	From −7 to +7

*BVRT, Benton Visual Retention Test; B/A ratio, TMT B time divided to TMT A time; PVFT, Phonemic Verbal Fluency Test; SVFT, Semantic Verbal Fluency Test*.

### Participants

Patients diagnosed with cSVD will be recruited among in-hospital patients (1st and 2nd Department of Neurology, University Clinic 3, Sechenov First Moscow State Medical University, Moscow). According to the Standards for Reporting Vascular Changes on Neuroimaging released in 2013, the neuroimaging characteristics of cSVD consist of WMH, recent small subcortical infarct, cerebral microbleeds, perivascular space, lacune of presumed vascular origin, and brain atrophy (4). The brain MRI of the patient will be assessed by trained radiologist.

Potential participants will be screened for eligibility criteria.

#### Inclusion Criteria

- Age between 60 and 75 years.
- Diagnosis of a cSVD—made on the basis of MRI and clinical features, according to current neuroimaging standards for research into small vessel disease (4) and current diagnostic criteria (7).
- Ability to follow the procedures of the study, fluent in Russian language—assessed by self-report.

#### Non-inclusion Criteria

- History of symptomatic stroke, brain tumor, or traumatic brain injury—assessed by clinical history.
- Hypothyroidism, B12 deficiency, HIV/AIDS, neurosyphilis, autoimmune, and connective tissue diseases—assessed by clinical history.

#### Exclusion Criteria

- History of psychiatric comorbidities and substance use disorder—assessed by self-report, clinical history, and the AUDIT.
- Significant vision loss (leading to difficulties with performing cognitive tasks)—assessed by self-report and clinical history.
- Presence of dementia—assessed by history and clinical examination.
- Movement disorders (e.g., tremor, dystonia) that slow down the speed of performing cognitive tasks—assessed by clinical examination.
- Obstructive sleep apnea syndrome—according to the criteria of International Classification of Sleep Disorders III (14).
- Restless legs syndrome—according to the criteria of the International Restless Legs Syndrome Study Group (63).
- Current intake of neuroleptics, benzodiazepines, selective serotonin reuptake inhibitors, and hypnotics—assessed by self-report and clinical history.

Before recruitment, the MDs will explain to each participant the aim and nature of the study, the main steps to be undertaken, the expected duration, the potential risks and benefits and any discomfort it may lead to, voluntary participation, withdrawal from the study at any time, and that withdrawal of consent will not affect his or her subsequent medical assistance and treatment. After eligibility assessment, potential participants will receive an information paper and a consent form. The consent form will be signed before any study procedures begin. The informed consent (IC) is compiled in accordance with the National Standard of the Russian Federation for Clinical investigations and good clinical practice (GOST R ISO 14155-2014) for the creation of IC for information transfer (64). The protocol of the study and the content of the IC have been approved by the local ethics committee of the I.M. Sechenov Moscow Medical University (no. 15-19/25.11.2019). The study has been registered in ClinicalTrials.gov Protocol Registration and Results System (Identifier: NCT04569643 29.09.2020). The scheduled time for patient recruitment is September 2021 to September 2024. This period will be extended if necessary.

The patients responding to the criteria of inclusion without conditions listed in part 2.2.2 will undergo the routine general clinical assessment and study procedures (nocturnal PSG, neuropsychological assessment, and brain MRI). Patients with identified apnea/hypopnea index (AHI) equal to or more than 5 will be excluded, as well as subjects with conditions mentioned in part 2.2.3. All enrolled patients will be given recommendation according to 2019 American College of Cardiology/American Heart Association Guideline on the Primary Prevention of Cardiovascular Disease (65) for the purpose to manage generally recognized risk factors for cardiovascular disease.

### Nocturnal PSG

Participants will undergo nocturnal stationary PSG (SOMNOscreen™, SOMNOmedics GmbH, Germany) at baseline. The PSG protocol will include electroencephalography (EEG) with registration of six monopolar recordings (Fp1A2, Fp2A1, C3A2, C4A1, O1A2, and O2A1); two electrooculography (EOG) channels; one electromyography (EMG) channel for chin muscles; two EMG channels for right and left tibialis anterior muscles; electrocardiography (ECG); recording of chest and abdominal movements, respiratory flow signal, blood oxygen saturation, and snoring; and registration of body position. Synchronized video recording will be made. Trained somnologist will analyze the data manually in accordance with criteria of The American Association of Sleep Medicine Manual for the scoring the sleep and associated events, version 2.6 (2020) (66). SOMNOwatch software “DOMINO® version 2.9.0” (SOMNOmedics GmbH, Germany) will be used for processing of records.

Sleep parameters will be calculated taking into account rules for recording and scoring periodic leg movements in sleep and wakefulness determined by the International Restless Legs Syndrome Study Group in 2006 (67) and then revised in 2016 (68). Following values will be obtained: total sleep time (TST), sleep-onset latency (SOL), wake after sleep onset (WASO), sleep efficiency (SE; percentage TST of time in bed), AHI, total number of LMs, number of LM per hour of sleep LM index (LMI), PLMI, index of periodic limb movements with arousals within 3 s of movement termination (PLMAI), periodic limb movements of wakefulness index (PLMWI), periodicity index (PI), and short-interval leg movements index (SILMS index).

Patients with obstructive AHI (equal to or more than five episodes per hour of sleep) will be excluded from the study and will be given recommendations for following examination and treatment.

Movements with a duration equal to or more than 0.5 s are regarded as leg movements during the sleep (LMs), and those with a duration of <10 s are candidate LMs (68). Two to four unilateral candidate LMs from the two legs overlapping each other within 0.5-s interval are referred to as one bilateral candidate LM if the total duration is <15 s. The PLMS are defined as candidate LMs series of four or more, separated by more than 5 and <90 s and not interrupted by LM > 10 s (68, 69). The total number of LMs during the sleep will be divided by TST to obtain LMI. The PLMI will be calculated as the number of PLMS per hour of sleep (70). Periodic limb movements during wakefulness (PLMW) are movements that meet the criteria of PLMS but occur during bedtime when patient is awake. PLMAI is a number of associations of PLMS series with EEG arousals per hour of sleep (71). The number of PLMW per hour of wakefulness during bedtime is PLMW index. The number of intermovement intervals that will be 10–90 s long and all in sequences of at least three will be divided by the total number of intervals to calculate the PI (70, 72). This index lies within 0 (absence of periodicity) to 1 (complete periodicity). Total number of doublets of LMs with intermovement interval <10 s is divided by TST to gain SILMS index (73). Experimental and control groups will be formed based on PLMI. The experimental group will include patients with PLMI ≥ 15/h, and the control group will include subjects with PLMI <15/h.

### Questionnaires

Self-reporting scales and questionnaires will be used to assess sleep quality, daytime functioning, and mood. The Pittsburgh Sleep Quality Index (PSQI) (74) is a self-report questionnaire that estimates sleep quality over a 1-month time interval. It includes seven components that assess different aspects of sleep quality, and each of components has a range of 0–3 points. The Global PSQI Score is the sum of the seven component scores, and it has a range of 0–21 points, where “0” reflects no difficulty and “21” reflects severe difficulties in all areas. The Russian version was approved by authors (75) and estimated in the Russian population (76).

The Insomnia Severity Index (ISI) is a brief self-reported tool measuring the perception of the subject of both nocturnal and diurnal symptoms of insomnia (77); the total score ranges from “0” (no symptoms) to “28” (severe symptoms). There is a Ph.D. research made by Rasskazova E.I. that contains a validation of the Russian version of the ISI (78).

The Epworth Sleepiness Scale (ESS) is a questionnaire that measures the subjective degree of sleepiness in common life situations. The patient is told to assess his or her chance to fall asleep in each of eight situations with a 4-point scale. The score of the ESS is the sum of these points; it ranges from 4 to 24 (79). The Russian version of the ESS was approved by the authors (80).

The Visual Analog Scale of Quality of Life (EQ-VAS), the part of EQ-5D, provides information about the health-related quality of life self-assessment of the patient (81). The Russian version of the EQ-5D is validated (82). The visual analog scale has 100 grades, where “100” represents the best imaginable health state, and “0” represents the worst imaginable health state (83). Geriatric Depression Scale (GDS) will be used for mood disorders assessment. It is a 30-item, self-report instrument that uses a “yes/no” format (84). GDS is useful for assessing patients with mild and moderate cognitive impairments, and its score ranges from 0 to 30; a greater score represents a greater risk of depression. The Russian version of the GDS is available and included in the Guidelines for Cognitive Impairment in Older adults approved by the Ministry of Health of the Russian Federation (85).

### Assessment of Cognitive Function

According to the “Diagnostic and Statistical Manual of Mental Disorders, Fifth Edition” (DSM-5), there are six domains of cognitive function: executive function, learning and memory, perceptual motor function, language, complex attention, and social cognition (86). Each domain has subdomains. Executive function consists of working memory, planning, inhibitory control, responding to feedback, flexibility, and decision making. Learning and memory include semantic and autobiographical long-term memory, free and clued recall, recognition memory, and implicit learning. Perceptual motor function is divided into visual perception, visuoconstructional reasoning, and perceptual motor coordination. Language as a cognitive domain includes the ability of grammar and syntax, object naming, fluency, word finding, and receptive language. Complex attention consists of sustained, divided and selective attention, and processing speed. Finally, social cognition includes recognition of emotions and theory of mind. Each domain except social cognition will be evaluated at baseline and after 2 years of follow-up. Both patient groups will receive identical test protocols.

Global cognition including learning and memory visual perception, visuoconstructional reasoning, executive functions, language, and complex attention will be assessed with the validated Russian version of the Montreal Cognitive Assessment (MoCA); the score ranges from 0 to 30 (87, 88). The Russian translation of the MoCA version 7.1 will be used (89).

For evaluation of learning and memory, the 12-word Verbal Memory Test and the Benton Visual Retention Test will be performed. The 12-word verbal memory test measures immediate and delayed recall of verbal material (a list of 12 nouns) using multiple list learning trials with a reminding paradigm (90). The score is the sum of immediate and delayed recall, and it ranges from 0 to 24. The Benton Visual Retention Test measures recall for 10 images with geometric designs; the first two images consist of one major geometric figure, and the other eight images are composed of two major geometric figures and a smaller peripheral figure (91). A multiple-choice administration will be used, when each image is showed for 10 s and then withdrawn; immediately after exposure, the patient is shown a multiple-choice card with four similar images. The subject should choose the identical one to the stimulus design image. The score of the Benton Test is the number of correctly recalled designs, and it ranges from 0 to 10.

The executive function domain will be measured with Frontal Assessment Battery (FAB) (92) ranging from 0 to 18, Trail Making Test (TMT) (93), parts A (the subject should draw a line linking consecutive numbers from 1 to 25) and B (the subject should draw a line linking alternating numbers and letters in sequence). Part B consists of numbers from 1 to 12 and the first 12 letters of the Russian alphabet. The time to complete each part of TMT is recorded, and the examiner will point out errors as they occur. The score is the time in seconds required for performing each part, and A/B ratio that reflects set shifting ability will be also estimated (94). The executive function is also estimated by phonetic verbal fluency test (naming nouns starting with a defined letter in 1 min), which is included in MoCA. The Russian version of the FAB is included in Guidelines for Cognitive Impairment in Older Adults approved by the Ministry of Health of the Russian Federation (85).

Complex attention and processing speed will be measured with the Symbol Digit Modalities Test (SDMT) (95), which is a subtest of the Wechsler Adult Intelligence Scale-Revised (WAIS-R) (96). The score is the correct number of substitutions in 90 s. Only the written response format of the SDMT will be conducted.

Language will be assessed with animal naming against the clock in 1 min (Semantic Verbal Fluency Test) that measures the ability to rapidly generate words from a specified category (97). The score of the task is the number of animals named.

### Neuroimaging

All subjects will undergo brain MRI scanning at baseline as a routine procedure and in the 2-year follow-up as a study procedure. The imaging protocol will include axial T1, T2, T2^*^, fluid attenuated inversion recovery (FLAIR), and diffusion weighted imaging (DWI) scans that will be made on a 3-Tesla scanner (Siemens, Erlangen, Germany). Slice thickness will be 5 mm, with an interslice gap of 1 mm and a matrix size of 192 × 256 pixels.

All the images will be processed with volBrain online software pipeline (98) to make an automated volumetric measurement of WMH. The volume of the white matter lesion will be calculated separately for the periventricular, juxtacortical, infratentorial, and deep white matter areas as well as the total volume of the lesion. Baseline volumes will be subtracted from follow-up volumes to obtain absolute WMH volume change. In addition, proportional volume change will be calculated by dividing the absolute WMH volume change by baseline WMH volume with a percentage display of results.

One trained and blinded rater will evaluate MRI scans with three cross-sectional visual scales [the Fazekas Scale (99), the Scheltens Scale (100), and the cSVD Score (101)] and the Rotterdam progression scale (102). The Fazekas Scale has a range of 0–6. Both in the periventricular and in the subcortical regions, scores 0–3 can be given for absence, mild, moderate, or severe lesions, respectively (99). The Scheltens Rating Scale ranges from 0 to 84: scores 0–6 can be given in 13 subcortical regions (frontal, parietal, temporal and occipital lobes, caudate, putamen, globus pallidus, thalamus, internal capsule, cerebellum, mesencephalon, pons, and medulla) and scores 0–2 for three periventricular regions (frontal caps, occipital caps, and lateral ventricles bands) (100). Change scores will be obtained by subtracting the baseline scores from the follow-up scores. Calculating the total SVD score (range 0–4) will be made by adding up of scores for each cSVD marker: 1 point for presence of any lacunes, 1 point for one or more cerebral microbleeds, 1 point for presence of enlarged perivascular spaces (PVS) in the basal ganglia, and 1 point for presence of moderate to severe PVS in other regions (101). The Rotterdam Progression Scale ranges from −7 to +7, measuring changes in WMH (−1, decrease; 0, no change; and +1, increase) for three periventricular regions (frontal caps, lateral bands, and occipital caps) and four subcortical regions (frontal, parietal, temporal, and occipital) (102).

### Outcomes

The primary outcome variable is total WMH volume change in percentage from baseline. The minimal clinically important change would be calculated based on baseline standard deviation as it was suggested by Lemieux et al. (103): the smallest effect size will be calculated by multiplying the baseline standard deviation by 0.2.

The secondary outcomes are the WMH volume change in each area of interests (periventricular, juxtacortical, infratentorial, and deep white matter), change in cognitive performance (results of each test will be processed separately) in percentage from baseline, cerebral infarct incidence, non-traumatic intracranial hemorrhage incidence, transient ischemic attack incidence, and rate of mortality due to stroke.

### Statistical Analysis and Sample Size Calculation

The baseline values of MRI scale scores and cognitive tests (listed in Table 1) will be subtracted from the corresponding follow-up values, and percent change from baseline will be calculated. Based on the PLMI (≥15/h or <15/h), two groups will be formed. In order to reproduce results of the cross-sectional study conducted by Kang et al. (51), group comparisons will be made using the Mann–Whitney test for non-parametric statistics or unpaired *t*-test, depending on whether the distribution is normal. To assess the association between the PLMI and total cerebral SVD sum score, as well as WMH volume, Fazekas Scale score, and Scheltens Scale separately, Spearman correlation analysis will be applied. To confirm the data obtained by Leng et al. (54) and complement it with a more detailed cognitive assessment, logistic regression will be utilized to study the association between PLMS and clinically significant changes in cognition from the baseline to the follow-up visit.

Multiple regression analysis will be used to estimate the capability of each of the characteristics of PLMS (LMs, LMI, PLMI, PI, and SILMS index) to predict each of the outcome variables (except for stroke incidence and mortality rate). Adjustment for main predictors of cardiovascular risk (age, hypertension level, body mass index, diabetes mellitus, ischemic heart disease, cholesterol level, and smoking) will be made to determine if the associations are independent of these factors. Comparison of stroke incidence (separately for cerebral infarct, non-traumatic intracranial hemorrhage, and transient ischemic attack) as well as mortality rate due to stroke between two groups will be made using Pearson's chi-square test.

Spearman correlation test will be applied to evaluate the association between the PLMI and scores of self-reported questionnaires (listed in Table 1) and assess the influence of PLMS on sleep quality, mood, and daytime functioning. The level of statistical significance will be set at *p* < 0.05. All statistical analyses will be performed using SPSS version 21.0 (SPSS Inc., Chicago, IL, USA).

Since the progression of cSVD features depending on PLMI is the key point of our study and linear regression will be applied, we calculated the sample size (N) based on this type of design. As mentioned above, there are seven independent variables (*p* = 7): age, arterial hypertension level, body mass index, blood sugar level, ischemic heart disease, cholesterol level, and smoking duration, and one tested parameter (PLMI or other parameters of PLMS). The sample size should be calculated depending on effect size: N = 392 + p for small, N = 52 + p for medium, and N = 22 + p for large effect size respectively (104). Thus, for revealing medium effect size, N = 52 + 7 = 59 participants should be enrolled, and for revealing small effect size, N = 392 + p, or 392 + 7 = 399 participants should be enrolled. A large effect size of PLMS is not expected. Therefore, 59 patients should be enrolled initially, and when no effect is revealed, the sample size should be enlarged up to 399. On the other hand, a comparison of cSVD feature prevalence between two groups is also planned as interim analysis. For this purpose, unpaired *t*-test (or Mann–Whitney test, depending on whether the distribution is normal) will be applied, and it requires standard deviation (SD) to calculate the sample size. When SD = 1.22 [obtained by Kang et al. (51)] and given probability for rejecting the null hypothesis α = 0.05, probability of failing to reject the null hypothesis under the alternative hypothesis β = 0.2 and effect size = 0.5, group size *N* = 49 is received when rounded up to the next highest integer. Therefore, at least 49^*^2 = 98 patients should be enrolled, but this number could be changed after another value of SD will be received.

## Discussion

PLMS are regarded as the possible sign of repetitive autonomic dysfunction during night sleep. Nevertheless, it is not known if this autonomic dysfunction can worsen the course of cardiovascular pathology, as well as increase the risk of cardiovascular events. In this regard, the investigation of the long-term effects of PLMS seems to be worth researching.

The proposed protocol of the study allows estimate influence of PLMS on both the course of cSVD and incidence of stroke. WMH increase and cognitive decline, which are the key features of cSVD progression, will be assessed. In comparison with the researches conducted by Kang et al. (51) and Del Brutto et al. (105), our study has a longitudinal observational design, which seems to be more appropriate for the purpose of risk factors identification than a cross-sectional design. These researchers came to the opposite conclusions in their studies that seem to support the utility of further investigation of the problem. Besides, the proposed protocol suggests a more detailed MRI assessment. Estimation of different brain regions separately is another strength of our study.

In comparison with another prototype study conducted by Leng et al. (54), the proposed study has also some strengths. The neuropsychological examination is more detailed and comprehensive. Particular attention is paid to the assessment of executive functions since they are affected by vascular cognitive impairment to a greater extent.

The results of the proposed research have potential practical value because PLMS is a treatable condition. If PLMS is identified as a predictor for cSVD progression, it could be regarded as a modifiable risk factor for cSVD, which can be managed. Eventually, it may contribute to improving patient outcomes.

Several limitations of the study should be considered. The design of the study does not allow to reveal the exact mechanism of how PLMS leads to cSVD progression and cognitive decline. If the hypothesis of the research is confirmed, we will be able to assume the possible underlying process only. Finally, because of the short follow-up period, no differences between groups might be yielded and the role of PLMS can be underestimated.

The reported association between high PLMI and transient sympathetic increase does not clarify whether it is a cause-and-effect connection or these events have the same origin. In this regard, more research using controlled trials is needed to demonstrate the positive impact of prolonged treatment of PLMS on cardiovascular health. The absence of a treated group is also one of the limitations of our study. It could establish the utility of pharmacological reduction PLMS.

Another significant limitation of the proposed study design should be discussed. The baseline level of both cognitive function and WMH volume determinates the speed of further progression of the cSVD. Therefore, the difference between groups at baseline will be the reason for the difference in disease progression. In order to deal with this problem, multiple regression analysis is applied and the baseline level will be included in multiple regression equation. Nevertheless, there is a potential bias related to the eligibility criteria. Only a subgroup of patients with AHI under 5 per hour will be included. Taking into account that the large proportion of cSVD patients has at least mild SOAS, the research will encompass only a small part of cSVD patients. However, we have to exclude even mild SOAS. Obstructive sleep apnea syndrome is accompanied by chronic sleep fragmentation as well as chronic hypoxemia that could worsen cognitive performance. Although the impact of mild SOAS on cognitive performance is still controversial (106–108), we cannot be sure that the presence of this condition will not influence the outcome. Taking into account that LMs in general and PLMS, in particular, could be associated with respiratory events, we would not include patients with even mild OSA with the purpose to avoid the presence of confounding factors.

Notwithstanding these limitations, the results of our study could elucidate the role of PLMS in cSVD progression and provide information about its possible influence on assessed outcomes—cognitive decline progression and MRI changes.

## Dissemination

Results of this trial will be disseminated via peer-reviewed journal publications. Primary endpoint results will be reported in a single publication. Other findings will be published separately. The results of this study will also be available on the ClinicalTrials.gov website.

## Author Contributions

ES, MP, and IF developed the study protocol and contributed to its description. ES and MP contributed to the eligibility criteria of the study and elaboration of the neuropsychology assessment. IF contributed to the sample size calculation and to the plan of statistical processing and defined details of PLMS assessment. All authors contributed to the conception and design of the study, article, and approved the submitted version.

## Funding

The study is supported by the scholarship program of German Academic Exchange Service (DAAD), funding program “Bi-nationally Supervised Doctoral Degrees/Cotutelle”, personal ref. no. 91775226, ID 57507869.

## Conflict of Interest

The authors declare that the research to be conducted has no commercial or financial relationships that could be construed as a potential conflict of interest.

## Publisher's Note

All claims expressed in this article are solely those of the authors and do not necessarily represent those of their affiliated organizations, or those of the publisher, the editors and the reviewers. Any product that may be evaluated in this article, or claim that may be made by its manufacturer, is not guaranteed or endorsed by the publisher.
